# Translations

**Published:** 1881-10

**Authors:** Seth N. Jordan

**Affiliations:** Columbus, Ga.


### Translations.

---

BY SETH N. JORDAN, M.D., COLUMBUS, GA.

From Foreign Journals for the ATLANTA MEDICAL REGISTER.

*Incision and Drainage of the Pericardium.* *S. Rosenstein, Wochenschrift, Berlin.*—In a boy ten years old a pericardial effusion was diagnosed, which the hypodermic syringe showed to be of a purulent character. A puncture with Protain's apparatus was made, but with only passing effect, as at this time a serous pleuritic extravasation associated itself with the original trouble. On this account an incision was made, under antiseptic precautions, between the fourth and fifth ribs and near the sternum. A large quantity of pus was evacuated, and the pericardium drained. It was found necessary to incise the pleura, also, as the extravasation in it had become purulent. Patient discharged, cured, in ten weeks. Author's conclusions: (1) Purulent pericarditis may run its course without fever and cutaneous œdema; (2) changes in the heart's sounds should not frighten us from operation; (3) when the pericardium contains large quantities of fluid, changes in the position of the recumbent body exercise no influence on the line of dullness.

---

*Spontaneous Rupture of the Stomach—Two Cases. By H. Chiari and L. Lantschner. Vienna Med. Blaetter.*—Chiari's case was that of a woman fifty-three years old, who died after having suffered from violent abdominal pains and repeated vomiting for several days. The post mortem showed the rupture to be through the mucous membrane, muscular coat and peritoneum, and through the scar of an old ulcer. There were several scars near this one, and also several slight ruptures in the mucous membrane. No erosion was observable. Subcutaneous emphysema developed at post mortem.*

The second case was also a woman, seventy years old. She had suffered from an umbilical hernia for forty-five years. After a walk she complained of great thirst, then of nausea, and afterwards began to vomit. During the vomiting a loud report was heard, which seemed to be inside the abdomen. Collapse, and death in thirteen hours. The autopsy proved the walls of the stomach to be ruptured and seemingly quite healthy.

---

*Alcohol in the Treatment of Polypus of the Ear. A Politzer, Vienna Med Blaetter.*—Politzer publishes two new cases of polypi of the ear recently successfully treated by him with alcohol. Not only to specialists, but also to the general practitioner, alcohol offers many advantages, according to P., in the treatment of all kinds of polypi, but especially in the following instances: (1) Where the root of the

*A case very similar to this one was reported in this paper, p. 906

polypus has its origin in the middle ear; (2) such as grow through Shrapnell's membrane; (3) where there is excessive proliferation of the mucous membrane of the middle ear, against which operations and cauterizations have been useless in almost every case; (4) such as have a broad base in the external ear; (5) when granulations are present on the drum; (6) in strictures of external ear; (7) when no anesthetic is given, and in children. The alcohol must be warmed slightly and poured into the ear three or four times a day. Length of treatment varies from two weeks to several months. Fibrous polypi often shrink up sooner than the soft granular variety.

---

*Diphtheria and Tracheotomy. By O. Pinner. Cases in Prof. Maas' Wards in Freiburg. Deutsche Zeitschrift fur Chirurgie.*—Pinner tabulates one hundred and one cases of tracheotomies, performed in Freiburg from April, 1877, to October, 1880. The difference between the number of boys and girls was very small. Recoveries 33, or 32.67 per cent. As to the influence of age, here again it was observed, as has been often experienced, that there are few chances when the operation is performed on children under two years old. Among those that recovered there was not one under two years. Most of the cases belonged to a later period in life, from two to six years. The greatest mortality took place on the first day after the operation; after the fifth day the prognosis became more favorable. From the rest of the article we learn that in the Freiburg Hospital the operation is performed with hanging head (Roser's position), tracheotomia inferior. The author speaks highly of moist warmth in the form of inhalations.

---

*Pilocarpin and Diphtheria. Neumeister, Deutsche Med. Wochenschrift*, 1881, *No.* 8.—The results obtained by Neumeister were altogether unfavorable. In the first place, salivation, especially among children, did not occur. Even in cases in which salivation followed its use, dissolution of the false membranes was not observed. Violent symptoms—collapse and feeble action of the heart—followed its use in several instances, and was each time directly attributable to the drug.

Of five adults treated in this manner, four recovered; of twenty-three children, thirteen died.

---

*Syphilis of the Lungs. Virchow's Archives, Von Cube.*—Patient was a man, thirty-four years old, who was infected with syphilis several years before, and for two years has suffered much with phthisical symptoms. The diagnosis of syphilis of the lungs was made, and based on the following facts: The disease was located in the middle and lower lobe of the right lung; apices intact; ulcerations in larynx. In addition, particles of tissue, which were found in the sputa, proved themselves of a gummous nature on microscopical examination. Patient recovered entirely after several inunctions with mercurial ointment.

---

*The Effects of Quinine on the Ear. P. Guder. Thesis. Berlin.*—From a series of experiments made by the author on healthy and diseased subjects, the following deductions were arrived at: 1 grm =15 grains quiniæ sulphatis lowers the tempearture of the ear about 0.57 Celsius=1° Fahrenheit; the time required is from two to two and a quarter hours. It diminishes the capacity for hearing the watch and for ordinary conversation in a marked degree. Following such doses of quinine, G. in no case observed a hyperæmia of the external ear and the drum, as is described by Roosa, but, on the contrary, parts which were at first congested, grew paler. Subjective aural sensations were perceptible in from one and a quarter to one and a half hours.

---

*Carbolism. R. Falkson. Wochenschrift.*—The following is a short resume of the article: Carbolic acid poisoning can be brought about by any "act" of Lister's method. The most productive source of all is washing out wounds that have deficient drainage with strong solutions of the acid. The gauze is the most innocent, but a simple compress saturated with a three per cent. solution is capable of introducing a considerable quantity of phenol into the system. The spray should be discarded as it promotes the resorption of acid through the wound, the skin and the lungs.

Anæmia, pyæmia, sepsis and especially nephritis, dispose to the resorption of the acid. Of all the tissues the peritoneum absorbs the acid the most readily, and after it follow the pleura, the serosa of the joints, and the bones. Symptoms of carbolism are, the characteristic green urine, headache, diarrhœa, sluggish pupils and fever. The fever is the most important symptom, not excepting the urine, as elevations of temperature take place before any changes are recognizable in the urine, or any other toxic symptoms are manifested. The elevations of temperature are striking, being of short duration, and occurring at every change of the dressing. As to treatment, karlsbad salts, recommended by Prof. Sonnenburg, was found of no avail. Large quantities of water assist the elimination of the acid in a marked degree, and water should thus be given as a prophylactic measure as well as an antidote.

---

*Carbolic Spasms. H. Treub. Centralblatt fur Chirurgie.*—Patient was a girl, eleven years old, suffering with an epipleural abscess. The abscess was opened with antiseptic precautions A few hours after the operation was performed slight symptoms of carbolism became evident, but disappeared again. On the sixth day twitchings of the whole body began, increasing in violence hourly, and accompanied by a feeble pulse, extremely shallow breathing, until finally these symptoms developed into an unconscious quasi moribund state. The carbolized dressings were removed, and the patient placed in a full bath. Immediately she began to regain consciousness. She was not entirely normal until the next day. The urine during the attack was quite green, and free from albumen. Afterwards the spray was used, causing a return of the poisoning, but only in a slight degree.

---

Naphthol is a new remedy recently recommended by Kaposi, of Vienna, for psoriasis. It is a product of tar, possessing healing virtues equal to chrysophanic acid, and at the same time being colorless and odorless, is strongly to be recommended. It also resembles chrysophanic acid in that it possesses toxic properties. A cumulative effect is to be feared when it is profusely used on large areas of psoriasis, which absorb with avidity. No unusual precautions are, however, demanded if the urine be regularly examined. Naphtol has also been recommended by Neisser for scabies, prurigo and kindred skin affections.

---

Thymol has been used by C. Bozzolo in 2–10 grm.=30–150 gr. as an efficient vermicide.

---

Friedberg, in Virchow's Archives, mentions a case of death from a dose of carbolic acid; quantity taken was 8.5 grm.=128 gr.

---

Hypodermic injections of 1 mmgr.=$\frac{1}{60}$ gr. of duboisine for exophthalmic goitre are used by Desnos, of Paris, with encouraging results.

---

In *Progres Medical* Boudet describes an apparatus for neuralgias. It is thus constructed: A tuning fork, mounted on a thin piece of wood 1 cmr. in diameter, is connected with a galvanic file. The fork is made to vibrate when it is ready for use. When placed on the supra-orbital region, analgesia and sometimes anæsthesia is produced in from eight to twenty minutes. It has been found of great benefit, notably in neuralgia of the trigeminus.

---

Crede, in the *Leipzig Maternity*, orders one drop of a two-per cent. solution of nitrate of silver to be distilled into the eyes of the new born, and compresses, steeped in a two-per cent. solution of salicylic acid, for the following twenty-four hours. Of two hundred children treated in this way, not one case of blennorrhœa developed.

---

THE *North Carolina Medical Journal* contains a report, carefully given, of removal of the inferior maxillary bone, through both rami, for osteoma-sarcomatous disease. Operation by Percy T. Norcop. Laryngotomy was performed to facilitate the operation. The patient died, after seventeen days, of capillary bronchitis, superinduced by exposure. Cases with like unfortunate termination often go unreported.

